# On Operations for Injuries of the Head

**Published:** 1863-01

**Authors:** A. Fisher

**Affiliations:** Chicago, Ill.


### CHICAGO MEDICAL EXAMINER.

N. S. DAVIS, M.D., Editor.

VOL. IV. JANUARY, 1863. NO. 1.

### Original Contributions.

### ARTICLE I.

### ON OPERATIONS FOR INJURIES OF THE HEAD.

By A. FISHER, M.D., of Chicago, Ill.

Read before the Chicago Medical Society.

In the treatment of injuries of the head, it is a great desideratum to understand the judicious use of the trephine, that we may secure to our patients all the benefits of an operation, when necessary, without subjecting them to the pain and danger of one that is unnecessary and, consequently, injurious.

Feeling the importance of being governed by correct principles, in designating such cases of injuries of the head as may be benefited by an operation, I shall endeavor this evening to call the attention of the Society to the subject, not expecting, however, to be able to say anything specially new in reference to it.

Before the time of the celebrated Doctor Pott, it was the practice of surgeons to trephine (or trepan as it was then called, from the name of the instrument used,) for almost all fractures of the skull, whether there were symptoms of compression or not. When there was fracture of the cranium with inflammation following it, they would trepan to give exit to the pus, before there was any evidence of its formation. If no inflammation had occurred, they would make a free opening with the trepan in different places along the fracture, for an outlet to all offending matters, as alleged, to prevent such an occurrence.

At one time the mania for trepanning was carried to such an extent, that surgeons seemed to vie with each other, to see which could bore the most holes in a patient's head, without killing him.

As an example of such practice, I will copy a certificate from LISTON'S Practice of Surgery, quoted from JOHN BELL'S Principles of Surgery :—

"I, the underwritten, Phillip, Count of Nausau, hereby declare and testify, that Mr. Henry Chadborn did trepan me in the skull twenty-seven times, and that after did cure me well and sound."

That a certificate should be procured to prove that the patient was cured "well and sound," after such an operation, is no wonder, especially as it is well known that the bone is never reproduced to any extent in the cranium, after the age of puberty. Thereby showing that the certificate of cure "well and sound," was as impossible and inconsistent as the operation was hazardous and useless. I have not alluded to the bygone practices of men, who were in their day at the head of the profession, for the purpose of ridicule, but merely to show the great improvement in surgery within the last half century.

At the present time, the principal indications for the use of the trephine in injuries of the head, are either to relieve the brain from undue pressure, or to remove pus, fragments of bone, or other foreign substances, from the substance of the brain, or from between the membranes covering it and the cranium.

Compression of the brain may be caused in various ways. We shall first speak of compression from extravasation of blood, caused by injuries of the head. To diagnose compression of the brain correctly, we must understand the symptoms of concussion as well as compression. In concussion, the patient is suddenly prostrated by the injury like a paroxysm of syncope; the pulse at first may be imperceptible or very indistinct, feeble, small, and frequent, respiration not much effected; the patient is generally sensible to external impressions, and dislikes to be disturbed; there is loss of memory or inability to comprehend, when apparently rational; the pupils are movable and not permanently dilated, though frequently insensible to the action of light; the extremities soon become cold, and the surface pale and contracted; before reaction is restored, the patient is almost sure to vomit, which is a favorable sign. In compression, there is complete insensibility, which may be sudden, or come on by degrees; the system is perfectly relaxed and insensible to external impressions; respiration is stertorous, slow, and laborious; pulse slow, full, and strong; pupils permanently dilated, and immovable by light; skin warmer than natural, and in bad cases, bedewed with perspiration; the bladder soon becomes distended, unless relieved by the catheter; the bowels move involuntarily.

The symptoms of concussion and compression are so very different that there is no difficulty in diagnosing compression at once, where it is uncomplicated with concussion. In most instances, however, when a patient receives an injury sufficiently severe to produce extravasation to any great extent, concussion either precedes or accompanies it, so that immediately after an injury we may have symptoms of both, commingled and changed in such a manner, that it is impossible to say how much the brain is suffering from compression alone. Although the symptoms of concussion may at first be well marked and unmistakable, without any sign of compression, still, we cannot be sure that extravasation will not occur when reaction is fully established. Therefore, in the treatment of concussion, it is well to bear in mind, that stimulants and other active measures calculated to bring on sudden reaction should be avoided; for, should the bloodvessels be lacerated, extravasation would be more likely to occur, and besides, the inflammatory symptoms would be aggravated by the means. It is generally sufficient in concussion, to apply warmth to the extremities, sinapisms over the stomach, and move the bowels with an enema: by such a course the vital powers will resume their functions more gradually, but just as surely, without the risk of a sudden reaction. When the system has recovered from the shock, we should endeavor in every possible way to prevent inflammation. Should there be extravasated blood to some extent, it may be absorbed, or the brain may accommodate itself to the pressure, if it is not too sudden, or the quantity of blood too great. When the brain is suffering from undue pressure from extravasation, whether sudden, or following concussion, and is not amenable to treatment, our hope is reduced to an operation, which unfortunately, is a dernier resort, that is generally very unpromising. Unless the injury is over a branch of the meningeal artery, with fracture, or of such a nature as to make it at least probable, that we can introduce the trephine directly over the ruptured vessels, our hopes of success are small indeed; even in the most favorable cases we are often disappointed, for frequently every thing will appear to locate the extravasation under the wound or injury, when perhaps, it will be found in the opposite side of the head, or somewhere out of reach. I have seen a number of instances of the kind revealed by *post mortem* examinations, after the trephine had been unsuccessfully used. Now, if there is so little hopes of locating the point of extravasation with certainty, the question arises: is an operation ever warranted in such cases? When the breathing is stertorous, pulse full, strong, and slow, with positive signs of compression, we ought to make an effort to save the life of the patient by an operation, if there is the least probability of ascertaining the location of the clot. For if we cannot relieve the brain from pressure, death is certain, and if we should succeed in removing the cause of compression, the patient may recover. But whether the operation is successful or not, we shall have the satisfaction of using all the means in our power to save life, with the consciousness that the operation would be harmless under the circumstances.

When there are evident signs of laceration of the brain, diagnosed by a frequent small pulse, cold extremities, and other symptoms of immediate collapse, no one in his senses would think of using the trephine. I have never known a patient recover from compression of the brain, whether operated on or not, whose pulse was 120 or more, after recovering from the preceding concussion.

Operations are sometimes required to give exit to pus accumulated between the cranium and dura mater, producing compression of the brain. The collection of pus in such cases, is the result of inflammation, either acute or chronic, which is generally caused by some kind of injury of the head, with or without, extravasation. The injury may be severe, or so slight as scarcely to be noticed at the time, the patient and even the surgeon may think that all danger has passed. But, perhaps, in a few days, or weeks, irregular chills and fever supervene, with pain in the head, the appetite fails, drowsiness comes on, with disinclination to talk or move. In short, we have all the symptoms of the formation of pus within the cranium. The symptoms growing more and more grave, until signs of compression are fully developed, which may be accomplished in a few days, or months may even pass, before the brain begins to suffer from undue pressure. Upon examining the head, if there is a wound it looks unhealthy, and does not heal kindly, the edges are generally pale, puffy, and glossy; the pus, if any is secreted, is not laudable, and perhaps, the cranium is denuded of its covering in the wound. In cases where there is no wound, there is generally a puffy swelling over the abscess, marking its location.

When we are fully satisfied, both from the constitutional and local symptoms, that there is a collection of pus between the dura mater and brain, and the constitutional symptoms are urgent, we should at once make an opening with the trephine for its outlet, more especially when there are signs of compression in addition. Notwithstanding we have every reason to believe, from the nature of the wound, and the constitutional symptoms, that the abscess is between the dura mater and cranium; we should be cautious in our diagnosis, for in some cases it may be found beneath the dura mater, in the substance of the brain, where an operation cannot reach it. Still the possibility of being mistaken in regard to the location of the abscess, ought not to deter us from giving the patient the benefit of an operation, when we have the ordinary signs to guide us; for if the pus is allowed to remain confined by the cranium, necrosis or death will surely follow.

In compound comminuted fractures, with displacement, operations are often advisable, when the brain is not suffering from compression. When the cranium is fractured, and a portion depressed and exposed to view by the injury, we should raise the depressed bone, or remove it entirely by using the trephine, if it cannot be raised without leaving comminuted pieces of bone to press upon, and irritate the dura mater; and the sooner the operation is performed (after the patient has sufficiently recovered from the shock,) the better, whether there is compression or not.

If we suffer the depressed bone to remain with its sharp edges, it will irritate, and perhaps, perforate the dura mater, and produce inflammation and suppuration of that membrane, with all its direful consequences. Then again, in all probability an operation will become indispensible, which, under the circumstances, will be more hazardous, and materially lessen the prospect of success. A case in illustration occurred in my practice a short time since:—

Mr. Mason, of a robust constitution, about 28 years old, whose residence is near Zanesville, Ohio, was stopping at the Brighton House, about five miles from the city, with a view of recruiting his horse, which he intended to offer for sale at the world's horse fair. When about to exhibit him to a friend, Sept. 1st, 1862, the day before the commencement of the fair, he was kicked by the horse on the side of his head. The toe caulk of the horse's shoe making a cut of a circular form, the convexity being upwards, three inches long, about an inch above, and a little anterior to, the top of the ear, on the left side of the head, making a smooth cut through the scalp, severing the posterior branch of the temporal artery, and fracturing the skull. A piece of the bone about two inches in length, longitudinally, and one inch vertically, was driven upon the dura mater and brain, besides comminuted pieces. Anteriorly, the bone was depressed about half an inch, and posteriorly, the thickness of the bone. I saw the patient about two hours after the injury: the attendants said that his extremities had been cold, but for the last half-hour were getting warmer. From the quantity of clot, I judged that he had lost 30 ounces of blood, or more. His pulse was weak and feeble, respiration normal, the extremities and surface generally, were rather cooler than natural: he was apparently insensible, but would move if handled. The symptoms were undoubtedly those of concussion, without compression of the brain. It was evident from the first view of the wound, that the depressed bones must be removed before the patient could recover. It was a question, however, of importance to decide, whether reaction was sufficiently developed to warrant the operation at that time. After mature reflection, I concluded that the shock of the operation would not depress the system as much as the irritation caused by the depressed bone, besides inflammation would be less likely to occur, to remove the depressed bone at once, than to defer the operation. I therefore shaved the head sufficiently, and ordered cloths wet in cold water to be applied, whilst I went home to make preparations to operate. Dr. MYERS returned with me, and administered chloroform to the patient, and assisted me in the operation. Although the patient was apparently insensible, he was in constant motion if touched, making it necessary to put him fully under the influence of chloroform, before we could operate. The bone being exposed by the injury, I had only to enlarge the wound sufficiently to introduce the trephine, we had to perforate the cranium in two places, before the depressed bone could be extracted without injury to the dura mater. After carefully removing every loose piece of bone, and waiting until the hemorrhage had entirely ceased, the parts were brought together and confined by sutures; a napkin wet in cold water was laid over the wound, with directions to keep it wet constantly. There being no compression before the operation, it produced no immediate relief. He was gradually recovering from the shock, and he continued to improve about the same after the operation. I prescribed no medicine, but left directions to keep him quiet, and give him nourishing drinks.

Sept. 2d. Found the patient improving, answers questions, and takes some nourishment, pulse 70, and regular, but weak; gave no medicine.

Sept. 3d. About the same, answers correctly when spoken to, but does not talk, or ask questions. Prescribed a cathartic of calomel and ipecac.

Sept. 4th. After the operation of the cathartic, this morning he aroused to perfect consciousness. He knew nothing about the injury, or any thing that had occurred after it, up to that time. Pulse 65, soft, and full, wound looks well, and is nearly healed; gave no medicine.

Sept. 6th. Removed the stitches. The wound is entirely healed, without a particle of suppuration, pulse natural: 65, soft, full, and regular. He has had no fever, or any inflammatory symptoms; and from that time I discontinued my visits; and his convalescence was rapid and perfect.

The favorable termination of the case just related is, in a measure, due to the great amount of blood that was lost immediately after the injury. In consequence of it, the vital powers were slow in resuming their functions, thereby preventing the occurrence of inflammation. The case shows the advantage in operating in compound comminuted fractures before inflammation supervenes, as well as the feasibility of uniting such wounds by adhesive inflammation. I am well aware that our best authors do not advise us to attempt union by the first intention, but in cases where the wound is a smooth cut, and all foreign substances can be removed, why we should not make the trial, I cannot imagine. For if we fail in accomplishing our object, no harm will be done; or should pus be formed beneath the scalp, we have only to make an opening to let it out, and that will be better than to have the whole wound open.

Although operations are generally advisable in compound comminuted fractures with depression, there are exceptional cases. When there is a slight wound of the scalp, fracture of the cranium, with one or more small pieces of it depressed, which do not irritate the dura mater, it will be best to defer the operation, and use every means in our power to prevent the occurrence of inflammation; but when in addition, we have the symptoms of compression, an operation should be performed without delay.

Punctured wounds of the head, with or without compression, generally require the use of the trephine immediately. It is difficult to penetrate the cranium with a pointed instrument, without comminuting, more or less, its hard brittle inner table, and if the broken pieces are allowed to remain, they will surely produce inflammation and suppuration of the dura mater, and perhaps, of the brain. Consequently, we ought, as soon as possible after the injury, to remove all loose pieces of bone or other foreign substances, with or without the trephine, as the case may require, whether the brain is suffering from compression or not.

Gun-shot wounds of the head are of a similar character, only more grave, requiring operations on the same principles.

Fractures of the cranium, with or without depression, where there is no solution of continuity of the soft parts, seldom require an operation, where there is no compression. Authors agree now a days, and experience has demonstrated, that when the cranium is fractured, and even comminuted and depressed to some extent, without injury or laceration of the scalp, that operations are not advisable, unless there is serious compression. I have seen many cases, where the cranium was fractured into a number of pieces, and more or less depressed, so that they could be felt moving under the finger, in which, recovery was perfect, without a sign of inflammation. Even though there should be some compression, the same course should at first be followed. But when the symptoms of compression are grave, whether they occur at the time of the injury, or days after, we should proceed to operate.

It occasionally happens that the patient will apparently recover from such injuries with the loss of memory, or some other faculty of the mind. A case in point, came under my observation 20 years or more since:—

A man about 40 years of age, was in a machine shop, watching the revolutions of a cast-iron cylinder, 8 or 10 inches in diameter. The revolving force was so great that it burst the cylinder, a portion of it was thrown against the upper part of the forehead of the patient, producing a fracture of the os frontis, slightly depressing a piece of the bone, about one and a-half inches square, on the left side of the head, and within half an inch of the mesial line. The scalp was bruised but not lacerated; severe concussion was produced, without compression. He was judiciously treated, to prevent inflammation, and consequently, the vital powers were slow in resuming their functions, and in a few days his mind appeared to be restored. It was soon ascertained, however, that his memory was impaired, but it was supposed that it would be restored as he improved in strength. He would answer correctly when spoken to, but would ask the same questions over and over again. There being no inflammatory action to cause the disturbance of the mind, and as he did not improve at the end of 4 or 5 weeks, an operation was concluded upon; and immediately on raising the depressed bone, he recovered his memory, having no recollection whatever of the injury, or of anything that had occurred after it. That space of time will, probably, always remain a perfect blank to him. It is often the case that the memory is lost for two or three days after severe concussion. I have seen a number of instances of the kind, but in this case the operation demonstrates that the loss of memory was caused either by compression, or by irritation of the depressed bone.

In young children, operations for compression are seldom required. The bones of the head not being fully ossified in children, are more yielding and not so brittle, consequently, not easily fractured. When they are fractured and depressed, there is a resilience which has a tendency to restore them to their normal conditions; besides the brain is gradually enlarging by its natural growth, and the bones not being perfectly formed, will accommodate themselves more easily to the abnormal pressure. Notwithstanding, it occasionally happens that we have to operate on very young children, and I will relate a case in point:—

About nine years ago, when I resided in Akron, Ohio, I was called to Bristol, Wayne County, Ohio, to operate on a bright little girl, about four years old. She was kicked by a horse on the side of the head, fracturing and depressing nearly one-half of the parietal bone. The child was perfectly insensible, and had been in a comatose state for forty-eight hours, the integuments were lacerated, and the bone partially exposed. I dissected up the scalp, and found a piece of the bone about three inches long, and two inches wide, separated and depressed nearly half an inch at the lower border, and one-fourth of an inch above. I tried my best to raise the bone without using the trephine, but found it impossible. I then introduced it, in hopes to get under the bone with the lever, so as to replace it, but was foiled. The idea of removing so large a piece of bone from the cranium of a child of her age was terrible, but there was no other hope. I finally made another opening with the trephine, and removed it with smaller pieces, without injury to the dura mater. Upon raising the bone, she at once came to her senses, and in a short time was perfectly conscious; the scalp was brought together and confined by three or four stitches, and she recovered without an untoward symptom. I saw her uncle about two years since, who said that the open space was completely filled with bone, and that her health had been good ever since the operation.

We prove by this case the practicability of removing large portions of the cranium in very young children, when necessary, as well as the recuperative power of nature, in reproducing new bone in such cases.

In regard to the manner of performing operations for injuries of the head, I have very little to say. Those who undertake such operations, should thoroughly understand the principles on which they are conducted, and then by ordinary skill and ingenuity, we are ready to adopt the kind of operation best suited to the particular case. We should, however, be careful in introducing the trephine, or elevating the bone, not to injure the dura mater, and be sure to leave no sharp points, loose pieces of bone, blood, or anything foreign within the wound: its edges then should be brought together, and most authors say, left to suppurate. But, as before stated, I see no reason why we should not endeavor to heal the wound by the first intention, when practicable. Above all, after operations on the head especially, antiphlogistic course should be strictly pursued, until all fears of inflammation have passed. For, just in proportion as we succeed in combatting inflammatory action, after the brain is relieved from pressure by an operation, will be our success in saving our patient.

